# The effect of Piper laetispicum extract (EAE-P) during chronic unpredictable mild stress based on interrelationship of inflammatory cytokines, apoptosis cytokines and neurotrophin in the hippocampus

**DOI:** 10.1186/s12906-015-0747-8

**Published:** 2015-07-17

**Authors:** Hui Xie, Di Jin, Yun Kang, Xueru Shi, Hongrui Liu, Haixing Shen, Jian Chen, Macheng Yan, Juan Liu, ShengLi Pan

**Affiliations:** School of Pharmacy, Fudan University, Shanghai, 201203 China; Department of Pharmacognosy, College of Pharmacy, Jiamusi University, Jiamusi, 154007 China; Pharmaceutical Preparation Section, Punan Hospital, Shanghai, 200127 China

**Keywords:** *Piper laetispicum*, Piperaceae, Ethyl acetate extract, Antidepressant, Hippocampus

## Abstract

**Backgroud:**

The *Piper laetispicum* C.DC. (Piperaceae) is a traditionally used herb in China for invigorating circulation and reducing stasis, detumescence and analgesia, which is distributed in the southern part of China and the southeastern part of Asia. Previous studies demonstrated that the ethyl acetate extract (EAE-P) of *P. laetispicum* possesses a significant antidepressant-like effect at doses higher than 60 mg/kg in Kunming (KM) mice, and this effect was not due to an increase in locomotive activity.

**Methods:**

To research this mechanism, in the present study, the chronic unpredictable mild stress (CUMS) model in Sprague–Dawley rats was used to further elucidate behavioral changes and corresponding changes in inflammatory cytokines (TNF-α, IL-6, IL-10), apoptosis cytokines (P53, Bax, Bcl2, caspase-3) and neurotrophin (BDNF) in the hippocampus of EAE-P treatment animals.

**Results and conclusions:**

The results suggest that EAE-P is beneficial to the behavioral outcome of the CUMS model animals, and decreased amounts of inflammatory cytokine IL-6 contributed to the antidepressant-like activation of EAE-P in every dosage group (15, 30, 60 mg/kg). In the low dosage group, down-regulated apoptosis cytokine p53 is associated with EAE-P effect, but it is inflammatory cytokine TNF-α that is related to the effect of EAE-P in the high dosage group. Meanwhile, the P53-dependent antiapoptotic effect of EAE-P may not be through Bcl-2 and Bax modulation. Furthermore, EAE-P showed up-regulated expression of brain-derived neurotrophic factor (BDNF) mRNA and down-regulated apoptosis cytokine caspase-3 mRNA, which was the same change tendency as with Fluoxetine.

## Background

Depression is one of the most burdensome diseases, usually associated with acute and persistent symptoms leading to life quality impairment [1]. Extensive studies of herbal medicines potentially useful in the treatment of depression have been conducted. Many of them have been shown to have high efficacy and safety, such as *Hypericum perforatum* (St. John’s wort), *Panax ginseng*, *Piper methysticum*, *Paullinia cupana* var. *sorbilis* (Guaraná) and Chaihu-Shugan-San, etc. [2–6].

The *Piper laetispicum* C.DC. (Piperaceae) is an endemic, climbing, glabrous plant distributed in the southern part of China and the southeastern part of Asia where it is popularly known as Xiao Chang-feng, Shan Hu-jiao, and Ye Hu-jiao. It is traditionally used for invigorating circulation and reducing stasis, detumescence and analgesia in China [7]. Laetispicine (N-isobutyl- (3,4-methylendioxyphenyl)-2E, 4E, 9E-undecatrienoamide) was previously isolated and reported for its exertion of antidepressant activity and antinociceptive effects in mice [8]. We further demonstrated that the ethyl acetate extract of *P. laetispicum* (EAE-P) possesses a significantly antidepressant-like effect at doses higher than 60 mg/kg in KM mice and proved that this was not due to an increase in locomotive activity [9].

In this present study, the chronic unpredictable mild stress (CUMS) model in Sprague -Dawley rats was used to further elucidate the antidepressant-like effects and mechanism of EAE-P. CUMS, the most promising rodent model for depression, is further supported and widely used for preclinical testing and screening of antidepressants [10–12]. In the CUMS model, animals are subjected to a variety of mild stressors, which mimic chronic stressful life events, presented intermittently for prolonged periods of time, and result in a behavioral deficit anhedonia, a core symptom of human depression [10]. Furthermore, since the hippocampus is critical for stress, learning and memory processes in depression and in the antidepressant response to pharmaco therapy [13], the corresponding changes in inflammatory cytokines (TNF-α, IL-6, IL-10), apoptosis cytokines (P53, Bax, Bcl2, caspase-3) and neurotrophin (BDNF) in the hippocampus were evaluated along with the behavioral evaluation.

## Methods

### Animals and grouping

Adult male Sprague–Dawley rats weighing between 180 and 200 g were purchased from the Department of Experimental Animal Center of Fudan University. Rats were housed under a normal 12 h light/dark cycle with lights on at 7 a.m. Tap water and standard food pellets were available *ad libitum*. Ambient temperature and relative humidity were maintained at 22–25 °C and 55 ± 10 %, respectively. Prior to the test procedure, rats were acclimatized the laboratory for seven days. The experimental procedures were conducted in compliance with the National Institutes of Health Guide for Care and Use of the laboratory animals and were approved by the Local Bioethics Committee (School of Pharmacy, Fudan University, China; document number: SYXK2007-002). Every effort was made to minimize the number and suffering of the animals used.

After a seven-day environmental adaptation, 54 animals were randomly divided into six groups: non-stressed control group (abbreviation: Control); 20 mg/kg fluoxetine group (abbreviation: Fluoxetine); model group (abbreviation: Model); 15 mg/kg EAE-P group (abbreviation: 15 mg/kg); 30 mg/kg EAE-P group (abbreviation: 30 mg/kg); and 60 mg/kg EAE-P group (abbreviation: 60 mg/kg). Each group’s baseline of 1 % sucrose preference, motor activity, body weight, and food consumption were measured. There were no significant differences amongst the groups. The results are shown in Table 1. The control rats were kept undisturbed in their home cages during the entire period of treatment, receiving only ordinary facility care with daily support of food and water, except for the sucrose intake test. The animals in other groups were fed separately in single cages.

**Table 1:** The baseline of sucrose solution consumption, motor activity, body weight and food consumption

Groups	1 % Sucrose solution consumption (%)	Motor activity			Body weight (gram)	Food consumption (gram)
		Score locomotion (times)	Rearing frequencies (times)	Grooming time (seconds)		
control	76.44 ± 2.19	137.78 ± 11.28	22.11 ± 2.71	10.96 ± 2.02	263.17 ± 4.45	27.90 ± 1.31
Fluoxetine	77.46 ± 2.24	135.81 ± 7.64	26.00 ± 2.10	12.19 ± 4.01	266.83 ± 3.18	28.20 ± 1.72
model	77.37 ± 2.29	120.72 ± 8.97	23.00 ± 2.39	13.88 ± 1.97	256.08 ± 6.36	33.00 ± 0.47
15 mg/kg	78.68 ± 2.48	138.69 ± 11.00	26.14 ± 3.62	10.84 ± 1.85	258.14 ± 7.74	31.74 ± 1.17
30 mg/kg	76.70 ± 3.90	119.42 ± 17.12	20.17 ± 3.11	16.28 ± 5.23	251.35 ± 4.88	30.86 ± 1.44
60 mg/kg	78.40 ± 3.47	135.19 ± 8.54	28.29 ± 4.17	17.58 ± 1.65	265.27 ± 3.51	28.17 ± 1.65

Data are expressed as mean ± S.E.M.(n = 8–9). The statistical analysis was performed by analysis of variance (ANOVA) followed by Bonferroni’s test

### Chronic unpredictable mild stress (CUMS) mode

The chronic unpredictable mild stress protocol was adapted from Gamaro et al. [14]. The eight-week CUMS paradigm’s first 28 days are shown in Table 2; the following days repeat it. One of the stressors was used each day and only interrupted by the sucrose preference test. This paradigm was used for the animals in the Fluoxetine, Model, 15 mg/kg group, 30 mg/kg group and 60 mg/kg group.

**Table 2:** The unpredictable chronic mild stress protocol

1	Light during the night	15	24 h of damp sawdust
2	5 min swimming at 4 °C	16	15 min Shaker rocking 1 time/second
3	24 h of food deprivation	17	Light during the night
4	24 h of water deprivation	18	24 h of water deprivation
5	Light during the night	19	1 min clipping tail
6	15 min shaker rocking 1 time/second	20	2 h of restraint
7	24 h of food deprivation	21	5 min swimming at 45 °C
8	1 min clipping tail	22	2 h of restraint
9	2 h of restraint	23	1 min clipping tail
10	5 min swimming at 45 °C	24	24 h of damp sawdust
11	5 min swimming at 4 °C	25	24 h of water deprivation
12	5 min swimming at 45 °C	26	5 min swimming at 4 °C
13	15 min shaker rocking 1 time/second	27	24 h of damp sawdust
14	24 h of food deprivation	28	5 min swimming at 4 °C

### Plant material

The stems of *P. laetispicum* were collected in 2006, from Hainan Province, China. The plant was identified by Professor Sheng-li Pan, School of Pharmacy, Fudan University, where a voucher specimen (No.060812) of the plant material has been deposited for further reference.

Before use, the dried stems of *P. laetispicum* were stored at −20 °C in freezer.

### Extract and drugs

The dried stems of *P. laetispicum* (20 kg) were powdered and percolated with 95 % EtOH (Ethyl alcohol), and the solution was concentrated under reduced pressure to give crude extract (1510 g). The ethyl acetate extract (EAE-P) was obtained following the methods published by our group [9] [15]. The HPLC profile was the same with Xie et al. [9]. And the content of four major components in EAE-P – Chingchingenamide (1.24 mg/g), Laetispiamide A (12.67 mg/g), Laetispiamide B (1.25 mg/g) and Laetispicine (9.65 mg/g) - were approached.

The EAE-P was kept refrigerated at + 4 °C and suspended in a solution of 2 % Tween-80 in saline by sonication with an ultrasonic cleaner (SK2200H, Shanghai KUDOS Ultrasonics instrument Co., Ltd (Shanghai, China)) less than 60 °C. The 2 % Tween-80 in saline also served as a solution control. Pharmacological screenings were performed and administered in doses of 15, 30 and 60 mg/kg body weight. All dosages were expressed as milligrams per kilogram body weight.

Fluoxetine-HCl, which purity was 98 %, was used in a concentration of 20 mg/kg body weight and prepared fresh on the day of administration in solution (2 % Tween-80 suspension in saline).

95 % EtOH, ethyl acetate, Tween-80 were purchased from Sinopharm Chemical Reagent Co., Ltd (Shanghai, China). Saline was from Shanghai Zhongxi Pharmaceutical Co., Ltd (Shanghai, China). Fluoxetine-HCl was from Cyber- Hubei Cyber Pharmaceutical Co., Ltd. (Wuhan, China).

### Sucrose preference test

A sucrose preference test was employed in order to determine anhedonia, one of the core symptoms of major depression in humans [11]. After environmental adaptation, rats were trained to consume 1 % (w/v) sucrose solution. Then the rats were simultaneously presented with 1 % sucrose solution and fresh water for 1 h after 23 h of food and water deprivation, as reported previously [16]. Sucrose preference (SP) was calculated according to the following formula: SP = sucrose intake/(sucrose intake + water intake) × 100 %. The sucrose preference test was performed each week and measured between 9 a.m. and 10 a.m. During this period, the animals were stressed daily, and the food and water were removed the night before the test.

### Open field test

The open field test method used in the present study was similar to that described previously by Kim [6]. The apparatus was a square arena (diameter: 80 cm; height: 40 cm) with a light source of 120 lx, which was demarcated into 25 equal areas. The score locomotion (number of line crossings), rearing frequencies (number of times an animal stood on its hind legs) and grooming time were recorded.

### Food utilization rate

Intake of food was measured by weighing the remaining food between 9 a.m. and 10 a.m. every day. Body weight was recorded at the end of each week. The food utilization rate (FUR) was calculated according to the following formula: FUR = body weight gain per week (g)/food-intake (g) per week × 100 % [17, 18].

### Blood collection and serum cytokine assay

After the last treatment of drugs, approximately 5 mL of blood was collected from the rat tail. Serum cytokine levels of BDNF were measured using the Rat BDNF (Brain-Derived Neurotrophic Factor) ELISA Kit (Xitang, Shanghai Xitang Technology Co., Ltd, Shanghai, China) and analyzed using Multiskan MK3 (Thermo, Thermo Scientific, USA). Serum from each animal was assayed in duplicate per manufacturer’s instructions.

### Real-time PCR

Total RNA was extracted from hippocampi using TRIzol reagent and assessed for quantity and integrity using agarose gel electrophoresis and Eppendorf Biophotometer Plus (Eppendorf, New Brunswick, CA). Total mRNA (1 μg) was reverse transcribed using PrimeScript^@^ 1-st Strand cDNA Synthesis Kit (TaKaRa, TAKARA Biotechnology (Dalian) Co., Ltd., Dalian, China) according to the manufacturer’s manual.

Real-time PCR was performed on a fluorescence ration PCR instrument (BioNeer, Korea) using SYBR^@^ Premix Ex TaqTM II (TaKaRa, TAKARA Biotechnology (Dalian) Co., Ltd., Dalian, China). Target cDNA (TNF-α, IL-6, IL-10, P53, BDNF, Bax, Bcl2, caspase-3) and endogenous control cDNA (actin) were amplified under the following conditions: 94 °C for 4 min, 35 cycles at 94 °C for 20 s, 60 °C for 30 s and 72 °C for 30 s. Relative quantitative (RQ) measurements of target gene levels were performed using the ΔΔCt method, where Ct is the threshold concentration [19]. The oligonucleotides used as primers are shown in Table 3.

**Table 3:** The oligonucleotides used as primers

Gene	Primer name	Sequence of primer	Target length
tnfα	rTNFaF	CTTCTCATTCCTGCTCGTGG	140 bp
	rTNFaR	ATCTGAGTGTGAGGGTCTGGG	
IL-6	rIL6F	AAGCCAGAGTCATTCAGAGCAA	160 bp
	rIL6R	TGGATGGTCTTGGTCCTTAGC	
IL-10	rIL10F	CCTGGTAGAAGTGATGCCCC	163 bp
	rIL10R	ATTCTTCACCTGCTCCACTGC	
AIF	rAIFF	CTGAGAAAGGAAATATGGGGAA	138 bp
	rAIFR	TGAGTAACTTGCCACCGCTG	
P53	rP53F	GGAAGGAAATCCGTATGCTGA	177 bp
	rP53R	GTGATGATGGTAAGGATGGGC	
Bax	rBaxF	CCCGAGAGGTCTTCTTCCG	167 bp
	rBaxR	GAAGTCCAGTGTCCAGCCCA	
caspase-3	rcaspase-3 F	CGAAACTCTTCATCATTCAGGC	129 bp
	rcaspase-3R	AGTAAGCATACAGGAAGTCGGC	
Bdnf	rBdnfF	CAGCGGCAGATAAAAAGACTG	187 bp
	rBdnfR	GTAGTTCGGCATTGCGAGTTC	
TrkB	rTrkBF	TATGAAGACTGGACCACGCC	167 bp
	rTrkBR	AGAAGCAGCATCACCAGCAG	
CREB	rCREBF	AGACCACTGATGGACAGCAGAT	150 bp
	rCREBR	TAGGAAGTGCTGGGGAGGAC	
Bcl-2	rBcl-2 F	GTGAACTGGGGGAGGATTGT	167 bp
	rBcl-2R	GCATCCCAGCCTCCGTTA	
actin	rat actin f	CCCATCTATGAGGGTTACGC	150 bp
	rat actin r	TTTAATGTCACGCACGATTTC	

### Western blot analysis

For immunoblotting studies, hippocampal total protein was prepared by homogenization in lysis buffer containing 20 mM Tris–HCl (pH 7.5), 150 mM NaCl, 1 mM EDTA, and 0.1 % sodium dodecyl-sulfate (SDS). The proteins were purified, resolved and electrotransferred as reported previously [20]. All blots were re-probed with β-actin antibody (1:4000 dilution, mouse monoclonal, Sigma, purchased from Sinopharm Chemical Reagent Co., Ltd (Shanghai, China)) as an internal control. Immunoreactive bands were revealed by an enhanced chemiluminescence kit (ECL Amersham from GE Healthcare) and detected using X-ray films. The immunoblot films were scanned, and the digitalized images analyzed with Gel-Pro Analyzer software.

### Statistical analysis of data

SPSS 15.0 was used for statistical analysis. Statistical significance between groups was performed by the application of analysis of variance ANOVA followed by Bonferroni’s test. Data obtained were expressed as mean ± standard error of the mean (S.E.M.). And P-values less than 0.05 (*p* < 0.05) were used as the significant level.

## Results and discussion

### Effects of EAE-P on Behavioral Tests in the CUMS-treated rats

**Fig. 1:**
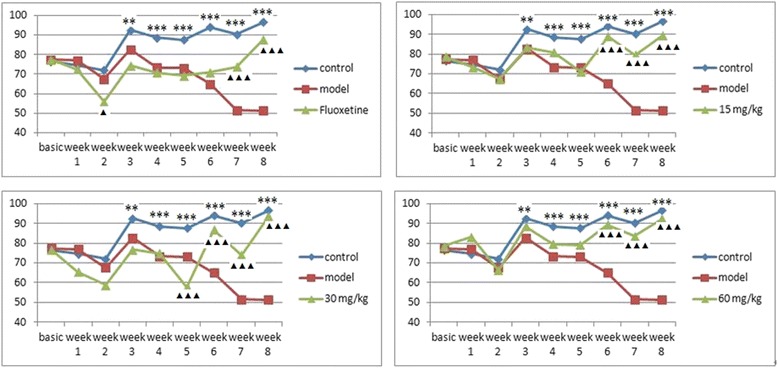
Effects of EAE-P on sucrose preference in the CUMS-treated rats. Results are expressed as mean ± S.E.M. (n = 7–9). Statistical analysis was performed by analysis of variance (ANOVA) followed by Bonferroni’s test. ▲ P <0.05, ▲▲P <0.01, **P <0.01, ▲▲▲P <0.001 and ***P <0.001 compared with model

The sucrose preference test was used to determine the animals’ depressive state [11]. As shown in Fig. 1, the baseline sucrose preference index was 76.44–78.68 % (the numerical value display in Table 1). At the end of the third week, significant differences were observed between the control and model groups, which are the key indicator of successful implementation of the CUMS model. After the administration of different concentrations of EAE-P for five weeks, the sucrose preferences of the 15 mg/kg, 30 mg/kg and 60 mg/kg groups were significantly higher than the model group (p < 0.001). Meanwhile, treatment with Fluoxetine also significantly increased the percentage of sucrose consumption as compared to the control group (p < 0.001), but this result needed six weeks of administration, one week longer than EAE-P.

Nonetheless, at the second week of Fluoxetine and the fifth week of 30 mg/kg EAE-P, the sucrose preference was significantly lower than the control group (p < 0.05 and p < 0.001). This was very interesting.

The Open Field Test (OFT) is commonly used to investigate locomotor activity, exploratory and depressive-like behaviors in experimental animals [21]. The times of score locomotion and rearing frequencies, and the seconds of grooming time were measured during the OFT at the end of the CUMS procedure. These details are shown in Table 4. After eight weeks, no significant differences were discovered. This suggests the EAE-P given in a subchronic treatment regime was beneficial for the behavioral outcome, which was not due to an increase in locomotive activity.

**Table 4:** Effects of EAE-P on the open field parameters recorded for 6 min in rats

Groups	Score Locomotion(times)		Rearing Frequencies(times)		Grooming Time(seconds)	
	Begin	After	Begin	After	Begin	After
control	137.78 ± 11.28	121.00 ± 12.41	22.11 ± 2.71	16.78 ± 2.11	10.96 ± 2.02	18.83 ± 5.04
Fluoxetine	135.81 ± 7.64	97.75 ± 16.71	26.00 ± 2.10	13.50 ± 3.15	12.19 ± 4.01	40.47 ± 8.82
model	120.72 ± 8.97	100.33 ± 14.18	23.00 ± 2.39	25.33 ± 4.37	13.88 ± 1.97	21.60 ± 5.55
15 mg/kg	138.69 ± 11.00	126.50 ± 15.02	26.14 ± 3.62	17.88 ± 2.96	10.84 ± 1.85	14.67 ± 6.12
30 mg/kg	119.42 ± 17.12	99.71 ± 13.20	20.17 ± 3.11	20.71 ± 2.62	16.28 ± 5.23	25.99 ± 5.74
60 mg/kg	135.19 ± 8.54	128.75 ± 16.07	28.29 ± 4.17	24.50 ± 3.18	17.58 ± 1.65	21.33 ± 4.15

Data are expressed as mean ± S.E.M. (n = 8–9). The statistical analysis was performed by analysis of variance (ANOVA) followed by Bonferroni’s test

**Fig. 2:**
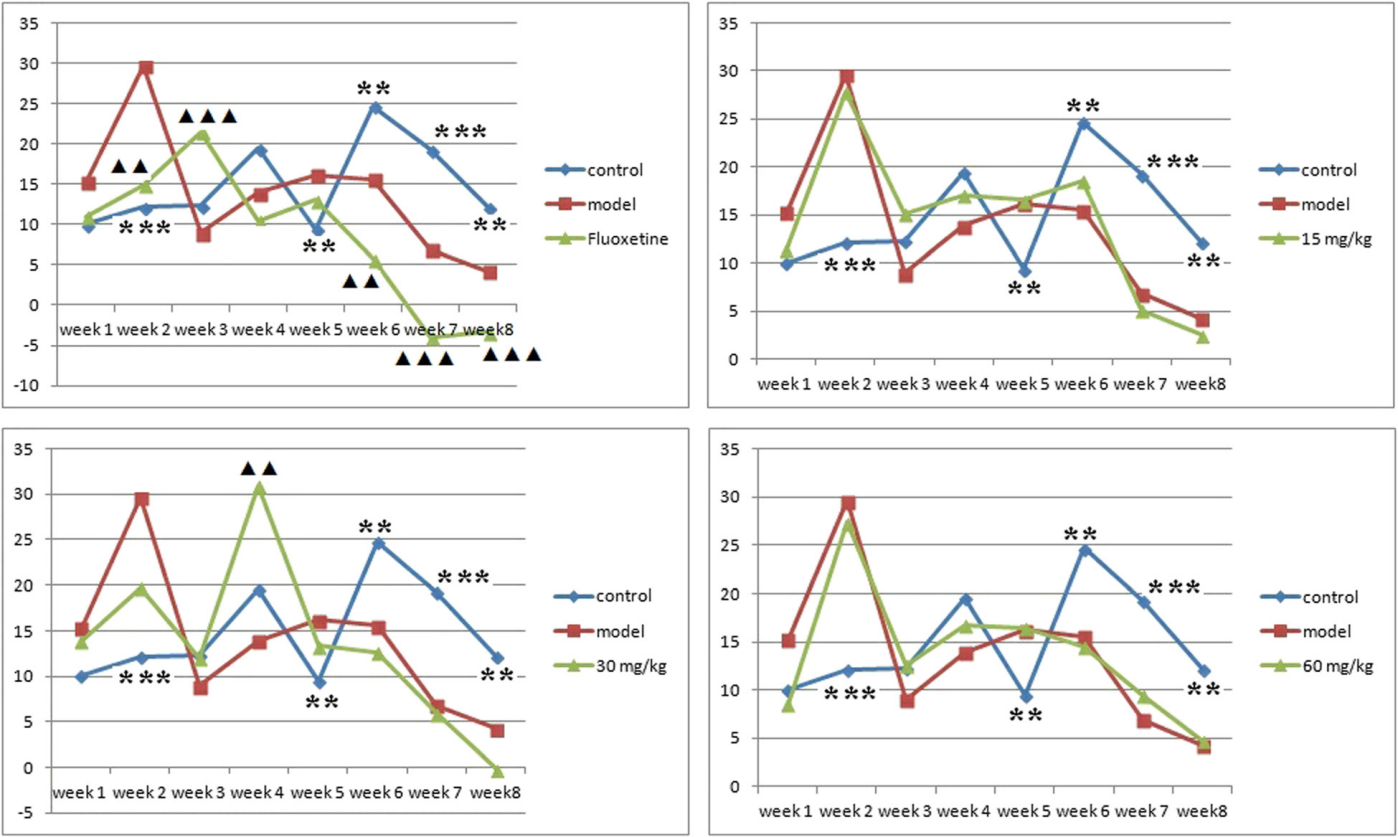
Effects of EAE-P on the food utilization rate (FUR) in the CUMS-treated rats. Results are expressed as mean ± S.E.M. (n = 7–9). Statistical analysis was performed by analysis of variance (ANOVA) followed by Bonferroni’s test. ▲▲P <0.01, **P <0.01, ▲▲▲P <0.001 and ***P <0.001 compared with model

For the Food Utilization Rate (FUR), as shown in Fig. 2, there were no significant differences amongst the groups during the first stressful week of the CUMS procedure. During the second week, the FUR of the model group and all of the EAE-P groups increased, and demonstrated significant differences compared to the control group (p < 0.001). At the end of the third week, the FUR was close to the control group, except for the Fluoxetine group, which was significantly higher than the model and control groups (p < 0.001). In the following week, the FUR of the 30 mg/kg group abruptly increased (compared to the other groups (p < 0.001)). At the next week (the fifth week), the FUR of the control group was unexpectedly lower than the model (p < 0.01) and 15 mg/kg (p < 0.05) groups. In the last three weeks, the FUR of the control group was significantly higher than the model and all of the EAE-P groups.

**Fig. 3:**
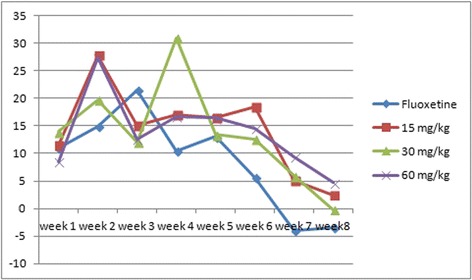
Comparision of food utilization rate (FUR) variation tendency between Fluoxetine group and EAE-P groups

Meanwhile, the Fluoxetine group was significantly lower than the model, 15 mg/kg or 60 mg/kg EAE-P groups at the second and the last three weeks, and significantly lower than the 30 mg/kg group at the fourth and seventh week. The details of comparison between the Fluoxetine group and EAE-P groups are shown in Table 5, which demonstrates that EAE-P may be more beneficial to the FUR than Fluoxetine. The variation tendency is shown in Fig. 3.

**Table 5:** Comparison of food utilization rate (FUR) between Fluoxetine group and EAE-P groups

Groups	Week 1	Week 2	Week 3	Week 4
Fluoxetine	11.03 ± 3.14	14.95 ± 4.42	21.39 ± 1.47	10.45 ± 0.88
15 mg/kg	11.44 ± 1.49	27.78 ± 3.99 ★	15.13 ± 1.37 ▲▲	17.07 ± 1.16
30 mg/kg	13.91 ± 2.55	19.74 ± 1.06	12.01 ± 1.47▲▲▲	30.88 ± 0.91★★★
60 mg/kg	8.53 ± 2.29	27.36 ± 1.69★★	12.61 ± 1.00▲▲▲	16.69 ± 1.37
Groups	Week 5	Week 6	Week 7	Week8
Fluoxetine	13.02 ± 1.09	5.70 ± 1.56	−3.99 ± 0.76	−3.37 ± 0.52
15 mg/kg	16.56 ± 0.84	18.52 ± 0.83★★★	5.15 ± 0.96★★★	2.49 ± 0.89★
30 mg/kg	13.39 ± 1.19	12.64 ± 1.29	5.92 ± 1.36★★★	−0.17 ± 0.54
60 mg/kg	16.41 ± 0.56	14.53 ± 1.68 ★	9.37 ± 1.75★★★	4.71 ± 0.72★★

Results are expressed as mean ± S.E.M. (n = 7–9). Statistical analysis was performed by analysis of variance (ANOVA) followed by Bonferroni’s test. ★★★ means the result is higher than Fluoxetine group. ▲▲▲means the result is lower than Fluoxetine group. ▲ p < 0.05,★p < 0.05, ▲▲P < 0.01, ★★P < 0.01, ▲▲▲P < 0.001 and ★★★P < 0.001 compared with model

In summary, EAE-P given in a subchronic treatment regime was beneficial to the behavioral outcome in an animal model of CUMS which induced depression-like behavioral changes.

### Effects of EAE-P on the inflammatory cytokines in CUMS-treated rats

In healthy individuals, there is a regulated balance between pro- and anti-inflammatory cytokines. Altered inflammatory cytokine profiles are often observed in depressed individuals [22].

**Fig. 4:**
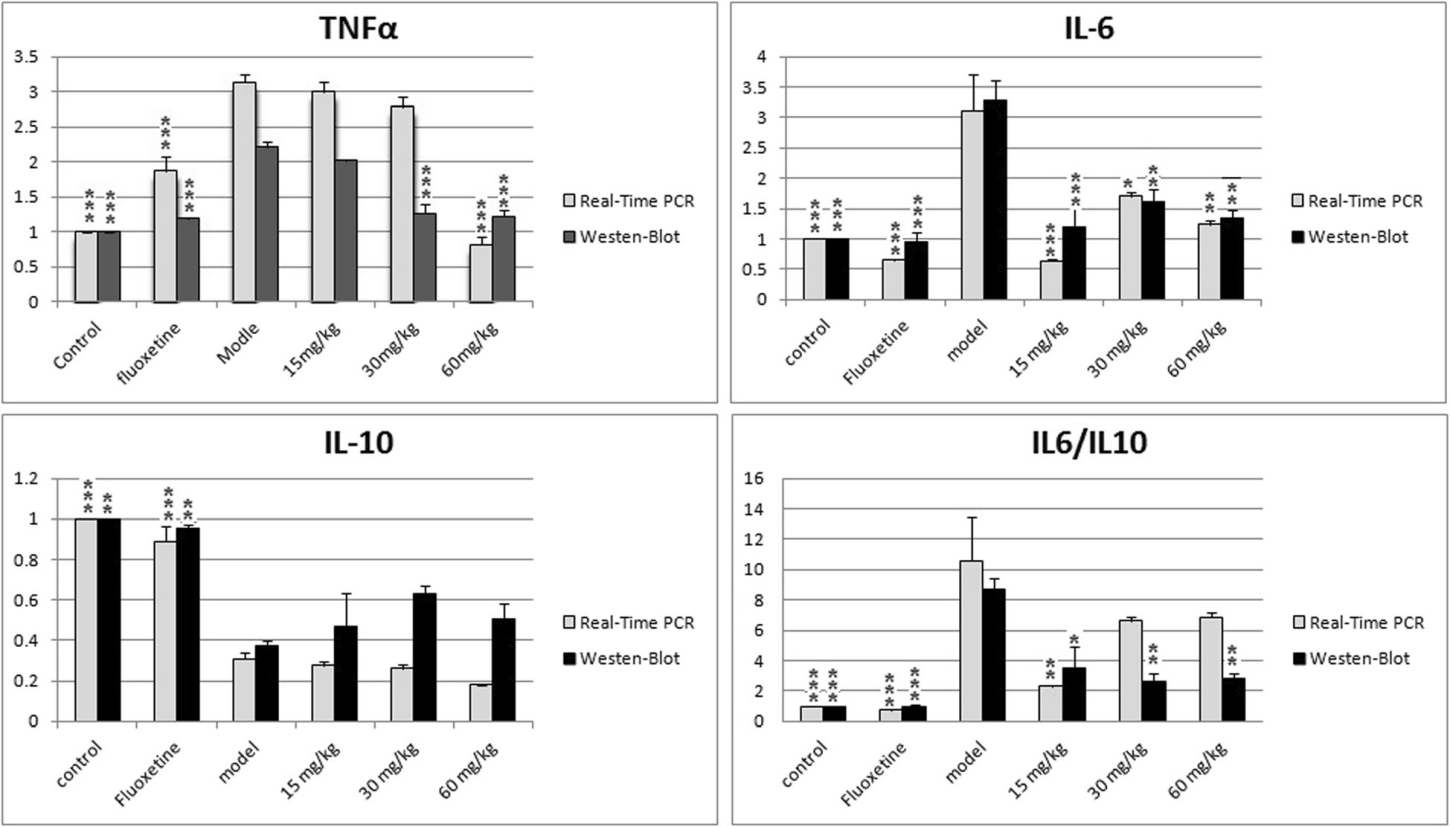
Effects of EAE-P on the inflammatory cytokines (TNF-α, IT-6, IT-10 and IL-6/IL-10 ratios) in CUMS-treated rats. Results are expressed as mean ± S.E.M.(n = 7-9). Statistical analysis was performed by analysis of variance (ANOVA) followed by Bonferroni’s test. * p < 0.05, ** p < 0.01, *** p < 0.001 compared with model

The tumor necrosis factor-alpha (TNF-α), which is a pro-inflammatory key signaling molecule, might contribute to the pathogenesis of depression because plasma levels of TNF-α and its soluble receptors have been found to be elevated in acutely depressed patients [23], and experimental stimulation of TNF-α production leads to depression-like emotional and cognitive disturbances in humans [24]. In the present study, the mRNA and protein expression level of TNF-α in the hippocampus was measured. Results show significantly higher in depressed subjects compared to controls (p < 0.001) (Fig. 4). Compared with the model group, Fluoxetine markedly (p < 0.001) reduced TNF-α mRNA and protein in the hippocampus. EAE-P treatment at 60 mg/kg also reduced the mRNA and protein (p < 0.001). However, EAE-P treatment at 30 mg/kg only reduced the protein expression but not mRNA. This revealed that a high concentration of EAE-P can effectively reverse the action of TNF-α.

In recent years, elevated interleukin (IL)-6 has been reported as a biomarker of depression [25]. IL-10 has been reported to play a role in regulating hypotha-lamic-pituitary-adrenal (HPA) axis homeostasis by suppressing adrenocorticotropic hormone-induced steroid production, and diminished IL-10 expression can affect HPA hyperactivity and glucocorticoid resistance seen in depressed patients [26]. In addition, the literature describes concurrent increases in IL-6 and decreases in IL-10 in individuals suffering from major depression. It is hypothesized that higher levels of depressive symptoms would be associated with lower IL-10 and higher IL-6 concentrations, as well as higher IL-6/IL-10 ratios. Also, as shown in Fig. 4, depressed subjects expressed significantly higher IL-6 mRNA and protein expression levels, lower IL-10 and significantly higher IL-6/IL-10 ratios compared to controls. Treatment at different concentrations of EAE-P, compared with the model group, showed significantly lower IL-6 (mRNA: 15 mg/kg, p < 0.001; 30 mg/kg, p < 0.05; 60 mg/kg, p < 0.01. Protein: 15 mg/kg, p < 0.001; 30 mg/kg and 60 mg/kg, p < 0.01), no significant change in IL-10 and partially significantly lower IL-6/IL-10 ratios (mRNA: 15 mg/kg, p < 0.001. Protein: 15 mg/kg, p < 0.05; 30 mg/kg and 60 mg/kg, p < 0.01). Meanwhile, the Fluoxetine group revealed significantly lower IL-6, significantly higher IL-10 and significantly lower IL-6/IL-10 ratios. These results indicate that the antidepressant-like activation of EAE-P is associated with inflammatory cytokine IL-6 and even IL-6/IL-10 ratios but not IL-10. That is different than the response to Fluoxetine.

### Effects of EAE-P on apoptosis cytokines in CUMS-treated rats

In addition to inflammatory cytokines, we also investigated the effect of EAE-P on mRNA expression of four apoptosis cytokines, because the apoptosis cytokines have been demonstrated by in vivo imaging studies that patients with major depressive disorders display reduced hippocampal and prefrontal cortex volume [27]. These structural alterations resulted from atrophy and loss of neurons and glia [28]. Activation of apoptotic pathways has been regarded as one of the most important way to cause these structural alterations. P53 is the “gatekeeper” of apoptosis. P53 activity is directed at its transcriptional target Bax [29], or it translocates to mitochondria to interact with Bcl-2 in a non-transcriptional way [30]. The caspase-dependent mitochondrial way is another approach.

**Fig. 5:**
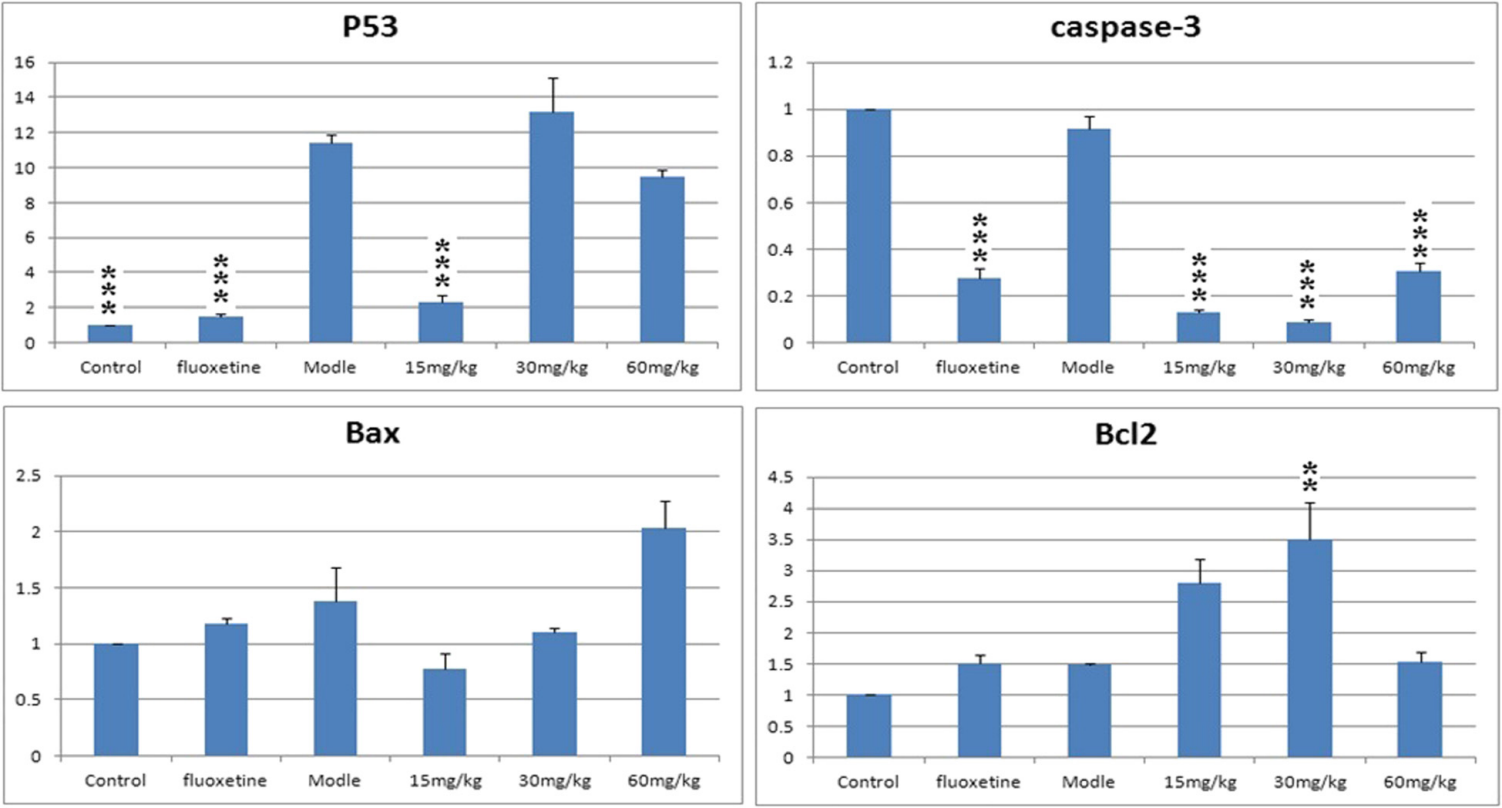
Effects of EAE-P on apoptosis cytokines (p53, Bax, Bcl-2 and caspase-3) in CUMS-treated rats. Results are expressed as mean ± S.E.M. (n = 7–9). Statistical analysis was performed by analysis of variance (ANOVA) followed by Bonferroni’s test. ** p < 0.01, *** p < 0.001 compared with model

In this study, p53 mRNA was upregulated by the CUMS treatment (Fig. 5). Fluoxetine and 15 mg/kg of EAE-P decreased hippocampal p53 mRNA expression (P < 0.001) when compared with the model group. This suggests that EAE-P pretreatment may depress p53 to inhibit apoptosis. The Real-Time PCR results of Bax and Bcl-2 showed that pretreatment with EAE-P did not produce pharmacological effects (Fig. 5), with the exception of Bcl-2 at 30 mg/kg. The influence of EAE-P on caspase-3 (Fig. 5) was showed that Fluoxetine and all the different concentrations of EAE-P markedly reduced caspase-3 mRNA expression compared to the control and model (P < 0.001), similar to the results obtained with Fluoxetine. Nevertheless, there is no significant difference in caspase-3 levels between the control and model. Further research is necessary to understand these results.

### Effects of EAE-P on the BDNF expression in CUMS-treated rats

The neurotrophin brain-derived neurotrophic factor (BDNF) is a member of the neurotrophin family and the most wide spread growth factor in the brain. It has diverse functions in the adult brain as a regulator of neuronal survival, fast synaptic transmission and activity-dependent synaptic plasticity [31]. Increasing evidence indicates that BDNF may play a role in the pathophysiology of depression and that antidepressants may, in part, exert their effects through regulation of BDNF. Decreased serum BDNF levels have been reported in depressed patients in several clinical studies, as has the fact that they can be normalized by antidepressant treatment [32]. Recent studies also have reported no effects or even increased BDNF levels in the hippocampus following exposure to chronic stress. It is possible that the effects of stress on BDNF mRNA expression in the hippocampus are dependent on several factors such as the type of stressor, the intensity, the duration, the frequency and the number of exposures [33].

**Fig. 6:**
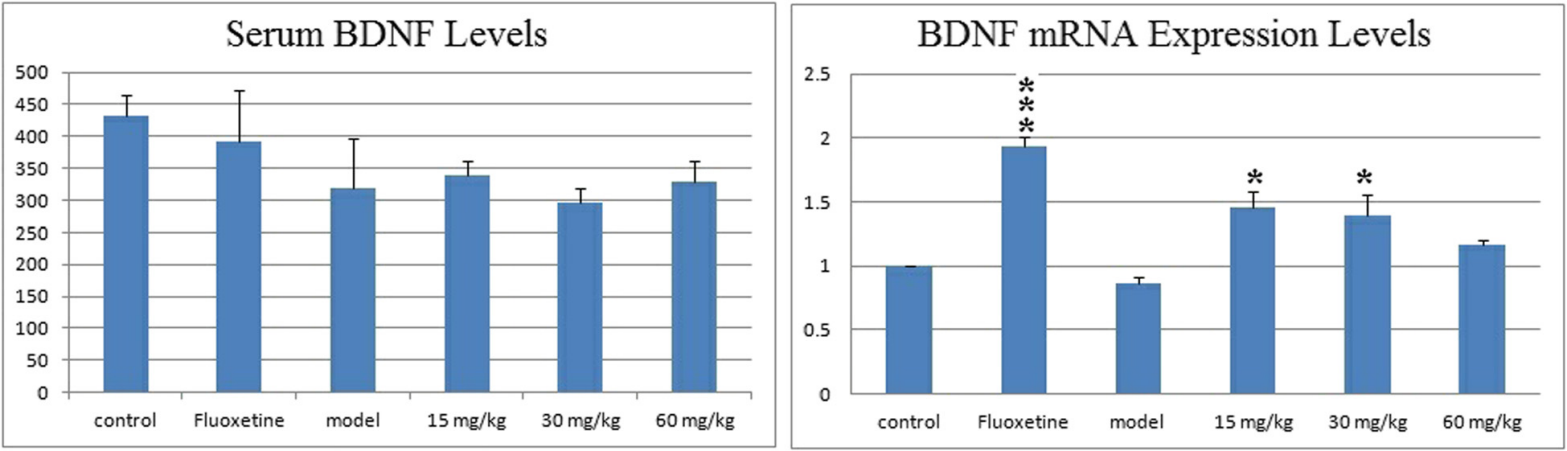
Effects of EAE-P on Neurotrophin (BDNF) in CUMS-treated rats. Results are expressed as mean ± S.E.M. (n = 7–9). Statistical analysis was performed by analysis of variance (ANOVA) followed by Bonferroni’s test. * p < 0.05, *** p < 0.001 compared with model

The levels of serum BDNF and the BDNF mRNA in the hippocampus are presented in Fig. 6. Although the level of serum BDNF and the BDNF mRNA expression are decreased in the model group compared to the control group, there are no significant differences between them. However, compared with the model group, Fluoxetine markedly (p < 0.001) elevated BDNF mRNA in the hippocampus. EAE-P treatment at 15 and 30 mg/kg also elevated it (p < 0.05). It has been suggested that BDNF levels may simply not correlate with depressive-like behavior, and a clear correlation between serum and brain BDNF levels still needs to be established. However, Fluoxetine and EAE-P showed up-regulated expression in this study, which needs a further research.

## Conclusion

The results reveal that EAE-P is beneficial to the behavioral outcome of the CUMS model animals, which was not due to an increase in locomotive activity. The decreased amounts of inflammatory cytokine IL-6 contributed to the antidepressant-like activation of EAE-P in every dosage group (15, 30, 60 mg/kg). However, in the low dosage group, down-regulated apoptosis cytokine p53 is associated with EAE-P effect, but it is inflammatory cytokine TNF-α that is related to the effect of EAE-P in the high dosage group. These suggest that the EAE-P needs to be subdivided for the further mechanism research. Meanwhile, the P53-dependent antiapoptotic effect of EAE-P may not be through Bcl-2 and Bax modulation.

Furthermore, EAE-P showed up-regulated expression of brain-derived neurotrophic factor (BDNF) mRNA and down-regulated apoptosis cytokine caspase-3 mRNA, which was the same change tendency as with Fluoxetine, although there is no significant differences between model and control in Sprague–Dawley rats’ CUMS mode.

## Abbreviations

EAE-P: Ethyl acetate extract of *P. laetispicum*
CUMS: Chronic unpredictable mild stress
OFT: Open field test
FUR: Food utilization rate
BDNF: Brain-derived neurotrophic factor
SP: Sucrose preference
